# Supplementary material for: Maternal antibiotic exposure enhances ILC2 activation in neonates via downregulation of IFN1 signaling
Source: Nat Commun. 2023 Dec 14;14:8332. doi: 10.1038/s41467-023-43903-x (PMC10721923; doi:10.1038/s41467-023-43903-x)
Supplement: Supplementary file 1 — Supplementary Information [file 41467_2023_43903_MOESM1_ESM.pdf]

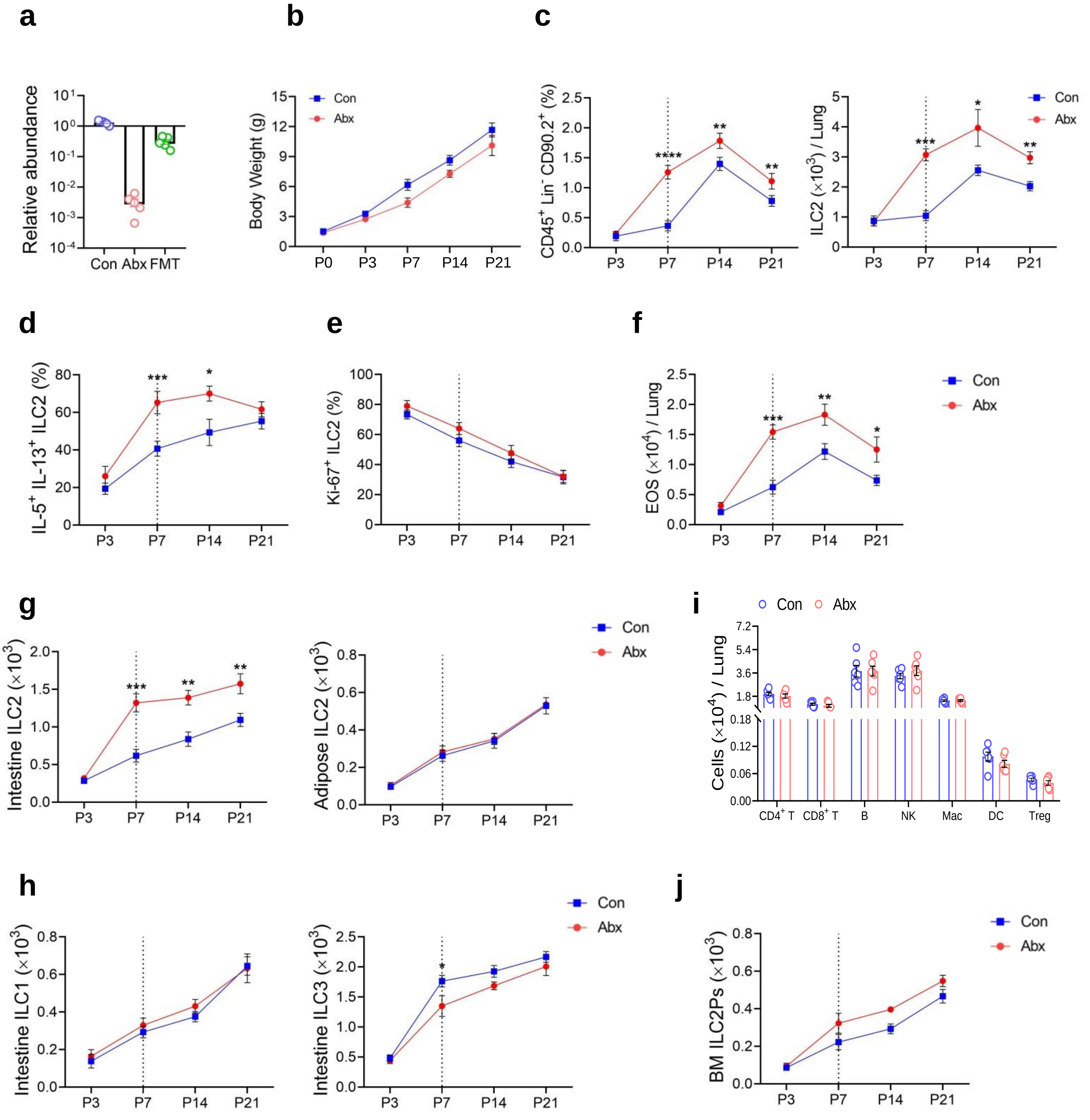

Supplementary Figure 1: Maternal antibiotic exposure affected ILC2 responses in neonates.

(a): The amounts of gut microbiota in the indicated groups was determined by qRT-PCR using universal 16S rRNA primers. **(b):** Body weights of offspring born to Abx-treated dams and the control dams. **(c-f):** The frequencies and absolute numbers of ILC2s **(c)**, IL-5$^+$ IL-13$^+$ ILC2s **(d)**, Ki-67$^+$ ILC2s **(e)** and eosinophils **(f)** in lungs of neonates born to Abx-treated dams and control dams. **(g):** The dynamic changes of ILC2 in in the intestines and adipose tissues of neonates born to Abx-treated dams and control dams. **(h):** The absolute numbers of ILC1(CD45$^+$Lin$^-$CD127$^+$T-bet$^+$) and ILC3(CD45$^+$Lin$^-$CD127$^+$RORγt$^+$) in the intestines of neonates born to Abx-treated dams and control dams. **(i):** The absolute cell counts of the indicated immune cells in neonatal lungs were analyzed by flow cytometry. **(j):** The absolute numbers of ILC2 progenitor in bone marrow was determined by flow cytometry. Data are shown as mean ± SEM(a-j).*P<0.05; **P<0.01; ***P<0.001; ****P <0.0001 by unpaired two-tailed Student's *t* test. Data are presentative of 2-3 independent experiments**.** Statistical source data are provided in Source Data.

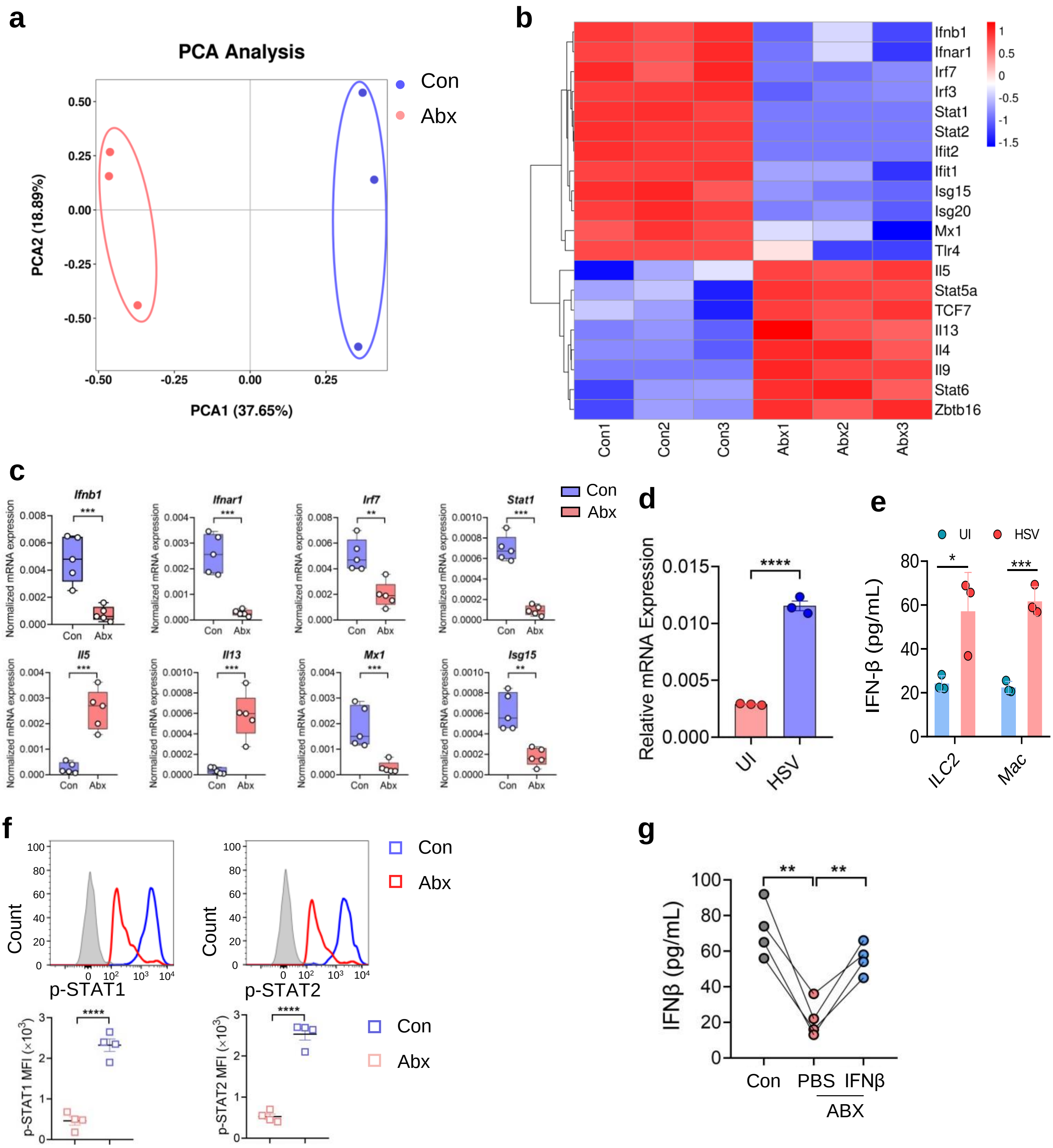

Supplementary Figure 2: Maternal antibiotic exposure downregulated IFN1 signaling in lung ILC2s from neonates.

(a): Principal Component Analysis (PCA) analysis of SMART-seq. **(b)**: Heatmap analysis of differentially expressed genes in SMART-seq. **(c):** The expression of IFN1 signaling genes was confirmed by qRT-PCR. **(d):**qRT-PCR analysis of *Ifnb1* mRNA in neonatal lung ILC2 treated with HSV (MOI, 10) for 12 hours (n=3). **(e):** Lung ILC2s and splenic macrophages from neonatal mice were infected with HSV (MOI, 10), the amounts of IFN-β in culture supernatants were determined by ELISA (n=3). **(f)**: The levels of STAT1/STAT2 phosphorylation was evaluated by flow cytometry in neonatal ILC2 from Abx-treated dams and the control dams. (**g**): Neonatal mice born of Abx dams were intraperitoneally injected with IFNβ (10000U/mice) once daily for 5 days, pups were sacrificed at P7. The amounts of IFNβ in homogenates of lungs were determined by ELISA. Neonatal mice from control dams were used as Control. Data are shown as mean ± SEM by unpaired two-tailed Student's t test**(d-g).** For box plots, the data are shown as "Min to Max, show all points". For box plots, the midline represents the median; box represents the interquartile range (IQR) between the first and third quartiles, and whiskers represent the lowest or highest values within 1.5 times IQR from the first or third quartiles(**c**). *P<0.05; **P<0.01; ****P <0.0001 by unpaired Student's *t* test. Data are presentative of 2-3 independent experiments. Statistical source data are provided in Source Data.

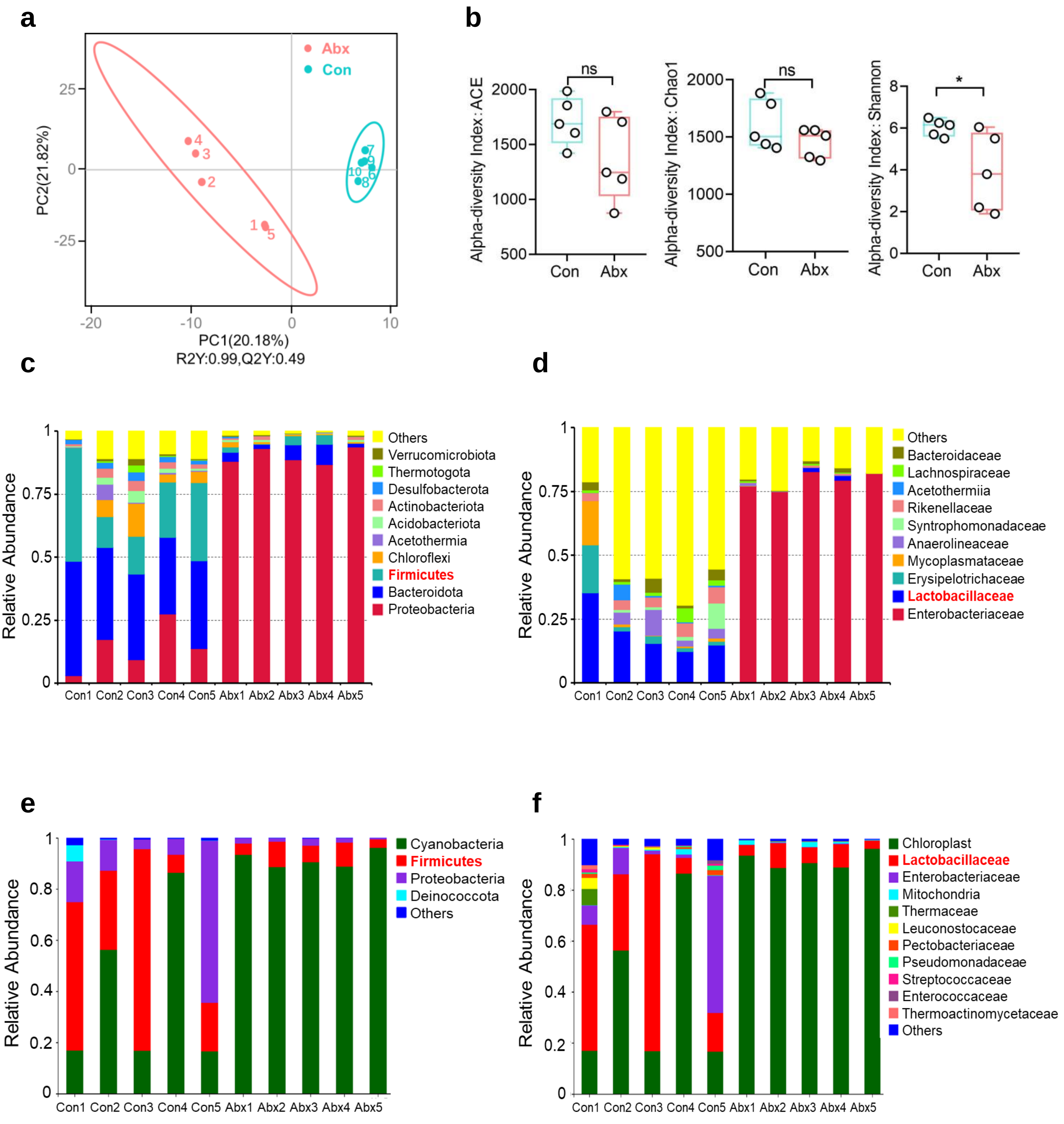

Supplementary Figure 3: Maternal antibiotic exposure modulated the composition of gut commensal bacteria in both dams and pups.

(a): Evaluation the composition of maternal gut microbiota by high-throughput 16S rRNA sequencing. β-diversity was shown as PLS-DA, which using the Bray-Curtis distance (n=5). **(b)**: α-diversity of the gut microbiota between the Abx dams and the control dams as indicated by the ACE, Chao1, and Shannon indices (n=5)**. (c-d):** Relative abundance of phylum level and family levels in dams (n=5). **(e-f)** Relative abundance of phylum level and family levels in pups (n=5). Data are shown as mean ± SEM; *P<0.05 by unpaired Student's *t* test**(b)**.

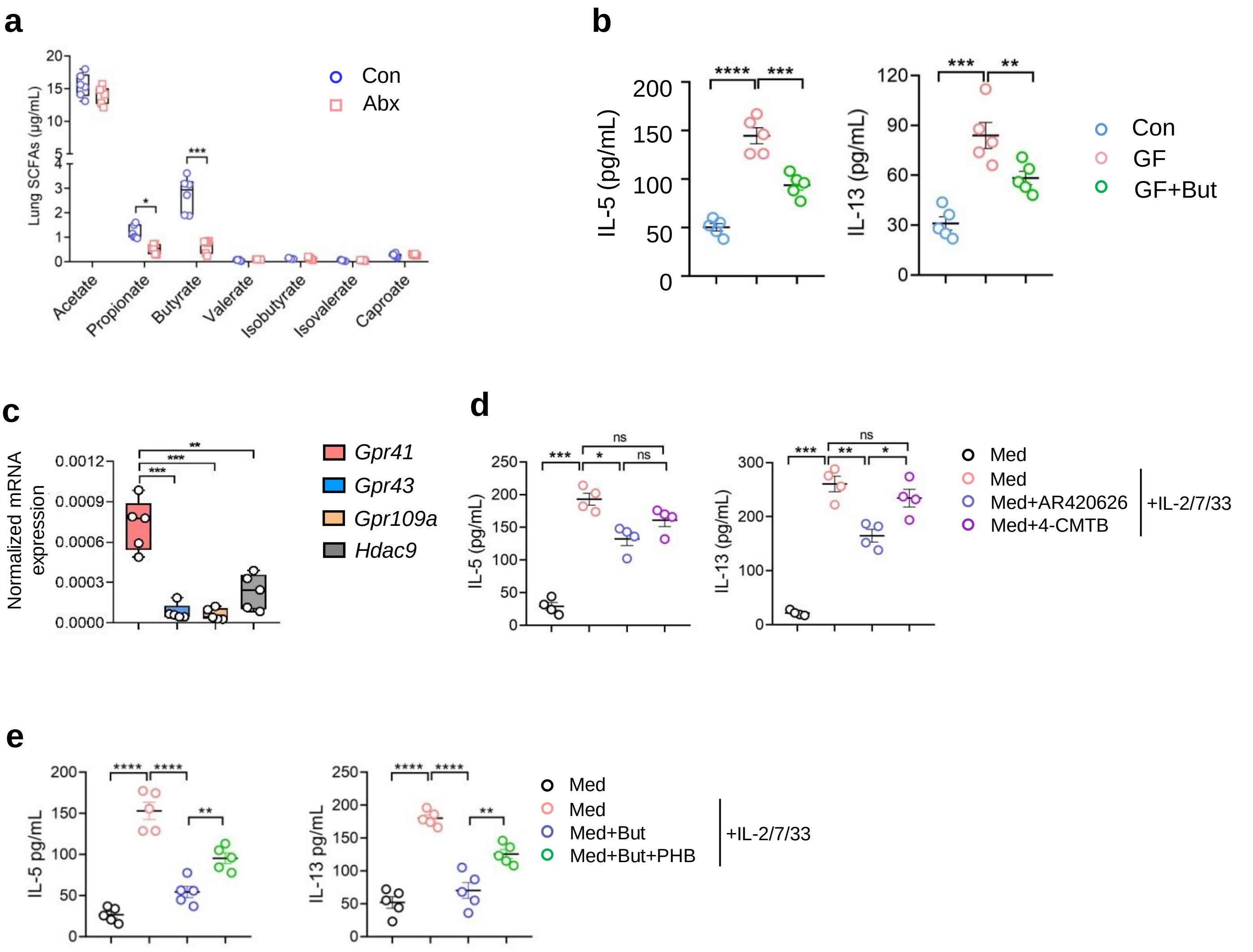

Supplementary Figure 4: GPR41 mediated the regulation of ILC2s by butyrate.

(a): Targeted SCFA metabolomics analysis by LC–MS in neonatal lung of Abx-treated dams and the control dams (n=6). **(b)**: GF newborn mice were intraperitoneally injected with butyrate (100 mg/kg) once daily for 5 days, pups were sacrificed at P7. The amounts of IL-5 and IL-13 in lung homogenates were examined by ELISA (n=5). **(c)**: mRNA expression of SCFA receptors in lung ILC2s from WT pups (P7) were evaluated by qRT-PCR (n=5). **(d)**: Lung ILC2s from WT pups were cultured in the presence of IL-2, IL-7, and IL-33 for 3 days and treated with GPR41 agonist AR420626 (1 mM) and GPR43 agonist 4-CMBT (0.5 mM). The amounts of IL-5 and IL-13 in supernatants were determined by ELISA (n=4). **(e)**: Cultured lung ILC2s from neonatal mice were treated with butyrate (2 mM) with or without GPR41 antagonist polyhydroxybutyrate (PHB) (10 μM). The amounts of IL-5 and IL-13 in the supernatant were determined by ELISA (n=5). Data are shown as mean ± SEM by unpaired two-tailed Student's *t* test**(b, d, e).** For box plots**(a, c)**, the data are shown as "Min to Max, show all points". The midline represents the median; box represents the interquartile range (IQR) between the first and third quartiles, and whiskers represent the lowest or highest values within 1.5 times IQR from the first or third quartiles.*P<0.05; **P<0.01; ***P <0.001 and ****P <0.0001 by unpaired two-tailed Student's *t* test**(c)** and Mann-Whitney U test **(a).** Data are presentative of 2-3 independent experiments**(b-e)**. Statistical source data are provided in Source Data.

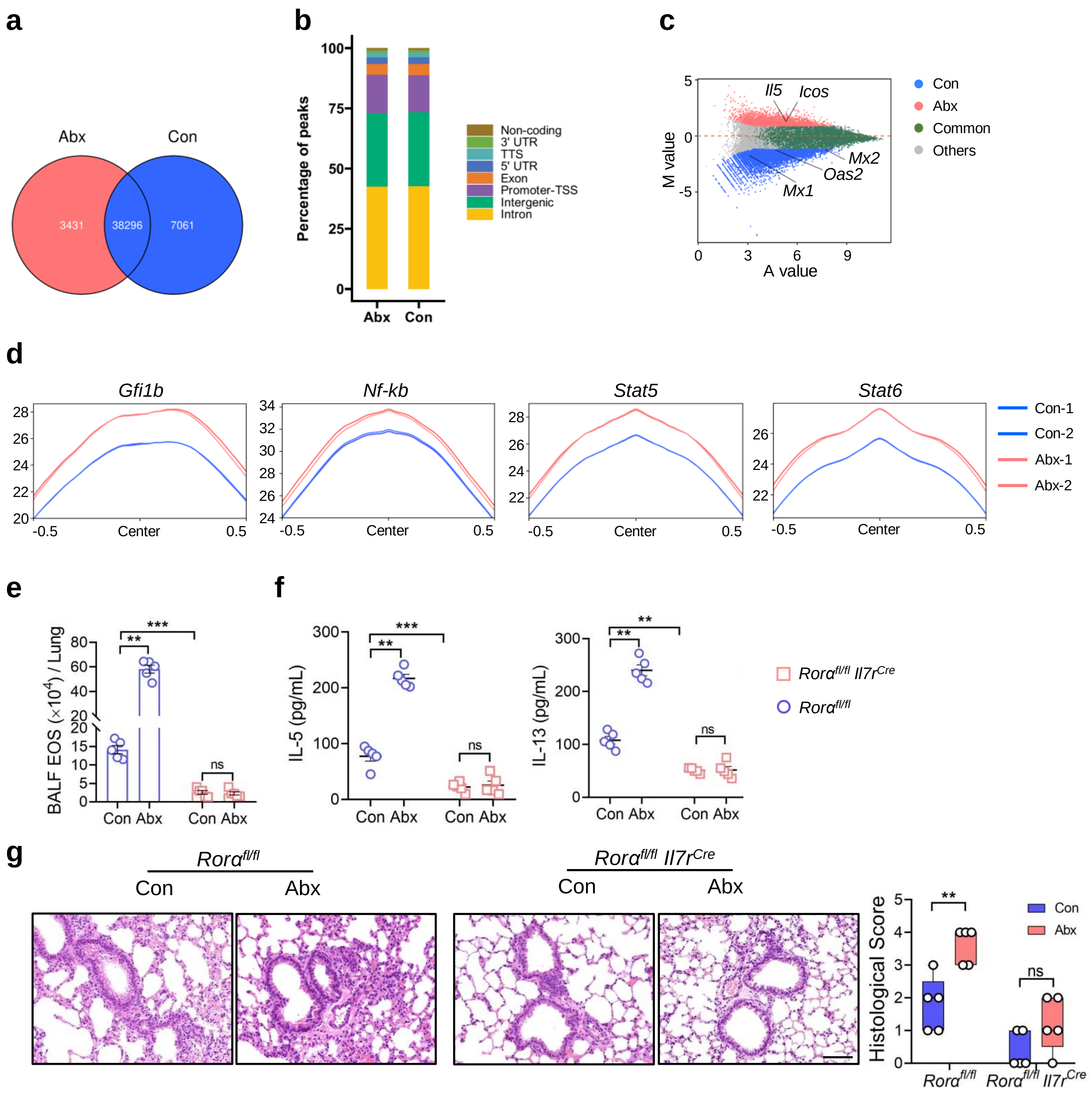

Supplementary Figure 5: Maternal antibiotic exposure induced epigenetic changes in ILC2s from adult offspring.

(a): Venn diagrams showing overlap of ATAC-seq peaks in ILC2s from Abx group (red) and control group (blue) (n=2). **(b)**: Distribution of ATAC-seq peaks across the genome in Abx and control group. **(c)**: Scatterplot represents the differences in chromatin accessibility between Con and Abx ILC2s. Red dots indicate peaks that are more accessible in Abx ILC2s, and blue dots indicate those that are uniquely accessible in control ILC2 (fold change > 2, FDR < 0.05). **(d)**: Transcription factor binding motifs of Gfi1b, Nf-kb, Stat5 and Stat6. **(e-g)**: *Rora*$^{fl/fl}$ *Il7r*^{Cre} or *Rora*$^{fl/fl}$ adult mice born to antibiotic-exposed dams or control dams were challenged with papain or PBS intranasally for 5 days; mice were sacrificed 24 h after the last challenge (n=5). **(e):** Frequencies of eosinophils in BALF were analyzed by flow cytometry. **(f):** Amounts of IL-5 and IL-13 in BALF were examined by ELISA. **(g):** H&E staining of lung tissues (bar, 100 μm) and histological score (n=5). Data are shown as mean ± SEM; *P<0.05; **P<0.01; ***P <0.001 by unpaired Student's *t* test or one-way ANOVA followed by Tukey-Kramer multiple-comparisons test**(e-f).** For box plots**(g)**, the data are shown as “Min to Max, show all points”. The midline represents the median; box represents the interquartile range (IQR) between the first and third quartiles. The top and bottom line represent the maximum and minimum values of the data. **P<0.01 by unpaired two-tailed Student's *t* test or one-way ANOVA followed by Tukey-Kramer multiple-comparisons test. Data are presentative of 2-3 independent experiments(e-g). Statistical source data are provided in Source Data.

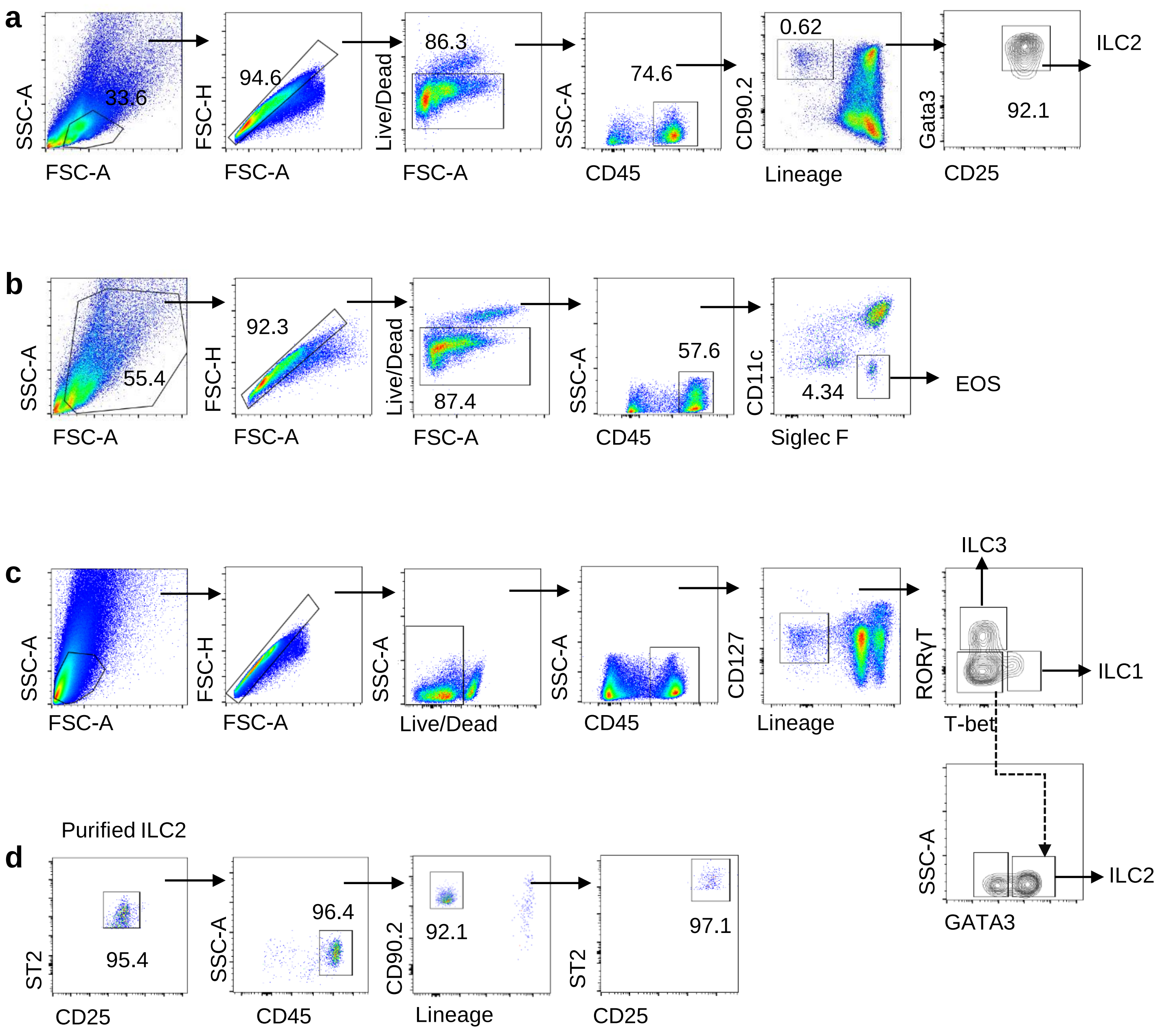

Supplementary Figure 6: Gating strategies for this paper.

(a): The gating strategy for neonatal lung ILC2. **(b):** The gating strategy for neonatal lung EOS. **(c):** The gating strategy for neonatal intestine ILCs, including ILC1s, ILC2s, ILC3s.**(d):** Purity of ILC2 at post-sorting.
