# Supplementary material for: Maternal antibiotic exposure enhances ILC2 activation in neonates via downregulation of IFN1 signaling
Source: Nat Commun. 2023 Dec 14;14:8332. doi: 10.1038/s41467-023-43903-x (PMC10721923; doi:10.1038/s41467-023-43903-x)
Supplement: Supplementary file 3 — Description of Additional Supplementary Files [file 41467_2023_43903_MOESM3_ESM.pdf]

Description of Additional Supplementary Files

Supplementary Data 1: Antibodies used for flow cytometry in this study.

Supplementary Data 2: Non-targeted metabolomic profiling.

Supplementary Data 3: SMART-seq data.

Supplementary Data 4: ATAC-seq data.

Supplementary Data 5: Sequences of primers used in this study.
