# Supplementary material for: Maternal antibiotic exposure enhances ILC2 activation in neonates via downregulation of IFN1 signaling
Source: Nat Commun. 2023 Dec 14;14:8332. doi: 10.1038/s41467-023-43903-x (PMC10721923; doi:10.1038/s41467-023-43903-x)
Supplement: Supplementary file 5 — Reporting Summary [file 41467_2023_43903_MOESM5_ESM.pdf]

nature portfolio

Corresponding author(s): Jie Zhou

Last updated by author(s): Oct 20, 2023

Reporting Summary

Nature Portfolio wishes to improve the reproducibility of the work that we publish. This form provides structure for consistency and transparency in reporting. For further information on Nature Portfolio policies, see our Editorial Policies and the Editorial Policy Checklist.

Statistics

For all statistical analyses, confirm that the following items are present in the figure legend, table legend, main text, or Methods section.

n/a	Confirmed	
☐	☒	The exact sample size (*n*) for each experimental group/condition, given as a discrete number and unit of measurement
☐	☒	A statement on whether measurements were taken from distinct samples or whether the same sample was measured repeatedly
☐	☒	The statistical test(s) used AND whether they are one- or two-sided<br>*Only common tests should be described solely by name; describe more complex techniques in the Methods section.*
☒	☐	A description of all covariates tested
☐	☒	A description of any assumptions or corrections, such as tests of normality and adjustment for multiple comparisons
☐	☒	A full description of the statistical parameters including central tendency (e.g. means) or other basic estimates (e.g. regression coefficient) AND variation (e.g. standard deviation) or associated estimates of uncertainty (e.g. confidence intervals)
☐	☒	For null hypothesis testing, the test statistic (e.g. *F*, *t*, *r*) with confidence intervals, effect sizes, degrees of freedom and *P* value noted<br>*Give P values as exact values whenever suitable.*
☒	☐	For Bayesian analysis, information on the choice of priors and Markov chain Monte Carlo settings
☒	☐	For hierarchical and complex designs, identification of the appropriate level for tests and full reporting of outcomes
☒	☐	Estimates of effect sizes (e.g. Cohen's *d*, Pearson's *r*), indicating how they were calculated

Our web collection on statistics for biologists contains articles on many of the points above.

Software and code

Policy information about availability of computer code

Data collection	1.qRT-PCR data were collected by the ABI 7500 Fast Dx instruments' Sequence Detection Software v2.0.5.<br>2.Flow cytometry was performed on a CytoFLEX S (Beckman Coulter) and data were analyzed using Flowjo V10.

Data analysis	1.Microsoft Excel 2007 and GraphPad Prism (8.0) for Statistical analysis and graph plotting.<br>2.In SMART-seq,the GO terms (http://www.geneontology.org/) and KEGG pathways (http://www.genome.jp/kegg/) of these differentially expressed genes were then annotated. GSEA was analyzed using a web tool (R package: clusterProfiler version 3.10.1) and mapped by gseaplot2 (R package: enrichplot version 1.2.0).<br>3.In Bacterial 16S rDNA amplicon and high-throughput sequencing, QIIME2 software was used to calculate the alpha diversity index and for beta diversity analysis.To determine the significance of differences in community structure between groups, the adonis and anosim functions in the QIIME2 software were used. To identify significantly different species at each taxonomic level (phylum, class, order, family, genus, species), R software (Version 3.5.3) was used for MetaStat and T-test analysis.<br>4. In Non-targeted metabolomic analysis, Partial least squares discriminant analysis (PLS-DA) was performed using metaX. Volcano plots were used to filter metabolites of interest based on log2 (fold change) and -log10 (P-value) of metabolites by ggplot2 in R language.<br>5. In ATAC-seq analysis, we annotated all peaks by using HOMER (version 4.11). As to pathway analysis, signaling pathway-related genes were chosen from the Molecular Signatures Database (MSigDB, version 7.5.1). Based on the commands bamCoverage (--binSize 10 --normalizeUding RPGC), computeMatrix (--upstream 1000 --downstream 1000), plotHeatmap, and plotProfile, deepTools (version 3.4.3) were applied to compare the chromatin open state around transcription start site (TSS).When it came to visualize the tracks, the Integrative Genomics Viewer (IGV, version 2.12.3) based on the 1x depth (reads per genome coverage, RPGC) normalization was used to make the signals from target genes between Abx group and the control comparable.

For manuscripts utilizing custom algorithms or software that are central to the research but not yet described in published literature, software must be made available to editors and reviewers. We strongly encourage code deposition in a community repository (e.g. GitHub). See the Nature Portfolio guidelines for submitting code & software for further information.

Data

Policy information about availability of data

All manuscripts must include a data availability statement. This statement should provide the following information, where applicable:

- Accession codes, unique identifiers, or web links for publicly available datasets
- A description of any restrictions on data availability
- For clinical datasets or third party data, please ensure that the statement adheres to our policy

SMART-seq (GSE231887) and ATAC-seq (GSE242246) data have been deposited at GEO and are publicly available as of the date of publication. Source data are provided with this paper in Source Data file. Any additional information required to reanalyze the data reported in this paper is available from the lead contact upon request.

Research involving human participants, their data, or biological material

Policy information about studies with human participants or human data. See also policy information about sex, gender (identity/presentation), and sexual orientation and race, ethnicity and racism.

Reporting on sex and gender	Not applicable
Reporting on race, ethnicity, or other socially relevant groupings	Not applicable
Population characteristics	Not applicable
Recruitment	Not applicable
Ethics oversight	Not applicable

Note that full information on the approval of the study protocol must also be provided in the manuscript.

Field-specific reporting

Please select the one below that is the best fit for your research. If you are not sure, read the appropriate sections before making your selection.

☒ Life sciences ☐ Behavioural & social sciences ☐ Ecological, evolutionary & environmental sciences

For a reference copy of the document with all sections, see nature.com/documents/nr-reporting-summary-flat.pdf

Life sciences study design

All studies must disclose on these points even when the disclosure is negative.

Sample size	For the animal experiments, sample size was determined based on pilot studies and previous experimental experiences. For cell and biochemical data, we aimed to collect data from three biological replicates when possible.
Data exclusions	No data were excluded from the analyses.
Replication	All attempts at replication were successful. How many times each experiment was performed and which statistical analysis was used is indicated in the figure legends.

Randomization	All samples were randomly allocated into experimental groups.
Blinding	The investigators were not blinded to the group allocation during data collection and/or data analysis. Data reported for mouse experiments are not subjective but rather based on quantitative flow cytometry.

Reporting for specific materials, systems and methods

We require information from authors about some types of materials, experimental systems and methods used in many studies. Here, indicate whether each material, system or method listed is relevant to your study. If you are not sure if a list item applies to your research, read the appropriate section before selecting a response.

Materials & experimental systems

n/a	Involved in the study
☐	☒ Antibodies
☒	☐ Eukaryotic cell lines
☒	☐ Palaeontology and archaeology
☐	☒ Animals and other organisms
☒	☐ Clinical data
☒	☐ Dual use research of concern
☒	☐ Plants

Methods

n/a	Involved in the study
☒	☐ ChIP-seq
☐	☒ Flow cytometry
☒	☐ MRI-based neuroimaging

Antibodies

Antibodies used

TCRβ-antibody, Clone:H57-597, Biotin, BioLegend;
NK1.1-antibody, Clone:PK136,Biotin, BioLegend;
CD4-antibody, Clone:GK1.5, Biotin, BioLegend;
CD11c-antibody, Clone:N418,Biotin,BioLegend;
CD5-antibody, Clone:53-7.3,Biotin, BioLegend;
CD8a-antibody, Clone:53-6.7,Biotin, BioLegend;
TER-119-antibody,Clone: TER-119,Biotin, Invitrogen;
CD11b-antibody,Clone:M1/70,Biotin, Invitrogen;
Ly-6G-antibody,Clone:RB6-8C5,Biotin, Invitrogen;
CD3e-antibody,Clone:17A2,Biotin, Invitrogen;
CD45R/B220, Clone:RA3-6B2,Biotin, Invitrogen;
Streptavidin,APC,Invitrogen;
Streptavidin,PE-CY7,Invitrogen;
Streptavidin,FITC,Invitrogen;
CD11c-antibody, Clone:N418,FITC, Invitrogen;
Siglec F-antibody, Clone:1RNM44N,PE,Invitrogen;
CD90.2-antibody, Clone:53-2.1,53-2.1,Invitrogen;
CD25-antibody, Clone:PC61.5,FITC/ PE-Cy7,Invitrogen;
ST2-antibody, Clone:DIH9,PE, BioLegend;
Ki-67-antibody, Clone:SolA15,FITC,Invitrogen;
IL-5-antibody, Clone:TRFK5,APC,BioLegend;
IL-13-antibody,Clone:eBio13A,PE,Invitrogen;
GPR41-antibody, Clone:1D10B7,Abcam;
Phospho-STAT1(Ser727)-antibody, Clone:A15158B,PE,BioLegend;
Phospho-STAT2 (Tyr690)-antibody, Clone:P52630,PE,BioLegend;
Mouse IgG1 Isotype, Clone:P52630,PE,BioLegend;
Live/Dead cell stain kit, KO525, ThermoFisher Scientific.

Validation

All commercially available antibodies were validated by vendors. Validation statements are provided on the manufacture's website. We examined primary antibodies according to manuals, and got similar results with validation results on manufacturer's website or relevant citations.

Animals and other research organisms

Policy information about studies involving animals; ARRIVE guidelines recommended for reporting animal research, and Sex and Gender in Research

Laboratory animals

C57BL/6 mice, the immunodeficient non-obese diabetic (NOD)-Prkdcem26Cd52Il2rgem26Cd22/Nju (NCG) mice, Germ-free (GF) C57BL/6J mice, Ifnar1-/- mice, ILC2 deficiency in Rorafl/fl Il7rCre mice and Gpr41-/- mice were maintained in specific pathogen-free conditions in the animal facility at Tianjin Medical University. Mice (male and female) were mated at 6-8 weeks of age. Pregnant mice were exposed to antibiotic cocktail (Abx) in the drinking water from embryonic days 10 to 14. Abx was then removed until birth. All

	mouse experiments were approved by the Institutional Animal Care and Use Committee of Tianjin Medical University.
Wild animals	The study did not involved wild animals.
Reporting on sex	Not involved.
Field-collected samples	The study did not involved samples collected from the field.
Ethics oversight	All mouse experiments were approved by the Institutional Animal Care and Use Committee of Tianjin Medical University.

Note that full information on the approval of the study protocol must also be provided in the manuscript.

Plants

Seed stocks	Not involved.
Novel plant genotypes	Not involved.
Authentication	Not involved.

Flow Cytometry

Plots

Confirm that:

- [x] The axis labels state the marker and fluorochrome used (e.g. CD4-FITC).
- [x] The axis scales are clearly visible. Include numbers along axes only for bottom left plot of group (a 'group' is an analysis of identical markers).
- [x] All plots are contour plots with outliers or pseudocolor plots.
- [x] A numerical value for number of cells or percentage (with statistics) is provided.

Methodology

Sample preparation	Single-cell suspensions were collected from lungs of adult mice as described previously. Protocols for neonatal mice were similar but modified slightly. In brief, lungs from pups at different days after birth were flushed by cold PBS three times via the right ventricle of the heart before removal. The lungs were then removed, cut into small pieces, and homogenized in cold PBS at 200 mg lung tissue/mL. After centrifugation at 1000 g for 20 min, the supernatant was collected and stored at -80°C for subsequent measurement. The pellet was resuspended in 3 mL collagenase type I (0.5 mg/mL in RPMI-1640 medium; Invitrogen) and incubated for 35 min at 37°C in a shaker at 200 rpm. To isolate cells from the lungs, the digested tissue was homogenized and filtered through a 70-μm cell strainer. Red blood cells were lysed in ammonium chloride–potassium (ACK) buffer.
Instrument	The instrument used for data collection was CytoFLEX S(Beckman Coulter).
Software	Flowjo (Version 10); Graphpad Prism (Version 8.0).
Cell population abundance	Purity of sorted population was more than 90% in all samples and was assessed by flow cytometry.
Gating strategy	All gate strategies captured cells by FSC vs SSC area, single cells by FSC height versus area. Gating strategies beyond this differed by experiment. Single-cell suspension was stained with different fluorophore-conjugated antibodies and analyzed by flow cytometry. ILC2s were gated as the Live&Dead-CD45+Lin-CD90.2+CD25+Gate3+ fraction.

- [x] Tick this box to confirm that a figure exemplifying the gating strategy is provided in the Supplementary Information.
